# Reservoir temperature prediction based on characterization of water chemistry data—case study of western Anatolia, Turkey

**DOI:** 10.1038/s41598-024-59409-5

**Published:** 2024-05-06

**Authors:** Haoxin Shi, Yanjun Zhang, Ziwang Yu, Yunxing Yang

**Affiliations:** College of Construction Engineering, Jilin University, Changchun, 130026 China

**Keywords:** Reservoir temperature, Hydrogeochemistry, Geothermometer, Machine learning, Environmental sciences, Solid Earth sciences, Geothermal energy

## Abstract

Reservoir temperature estimation is crucial for geothermal studies, but traditional methods are complex and uncertain. To address this, we collected 83 sets of water chemistry and reservoir temperature data and applied four machine learning algorithms. These models considered various input factors and underwent data preprocessing steps like null value imputation, normalization, and Pearson coefficient calculation. Cross-validation addressed data volume issues, and performance metrics were used for model evaluation. The results revealed that our machine learning models outperformed traditional fluid geothermometers. All machine learning models surpassed traditional methods. The XGBoost model, based on the F-3 combination, demonstrated the best prediction accuracy with an R^2^ of 0.9732, while the Bayesian ridge regression model using the F-4 combination had the lowest performance with an R^2^ of 0.8302. This study highlights the potential of machine learning for accurate reservoir temperature prediction, offering geothermal professionals a reliable tool for model selection and advancing our understanding of geothermal resources.

## Introduction

Geothermal energy is a renewable energy source that is rich in reserves, widely distributed, stable and reliable. Vigorous development and utilization of geothermal energy is of great significance to the implementation of carbon peak and carbon neutral goals. The determination of reservoir temperature is an important parameter indispensable to the study of geothermal resources exploration, geothermal potential assessment and geothermal development and utilization^1–5^. In order to make full use of geothermal resources, accurate prediction of reservoir temperature has become an active research topic^6^.

Generally speaking, the prediction methods of reservoir temperature can be divided into two categories: direct measurement method and indirect calculation method. The direct measurement method utilizes the site drilling to directly measure the temperature, and determines the thermal reservoir temperature by calculating the average of the thermal reservoir top plate and bottom plate temperatures. However, in most cases, there is no drilling hole in the site or the depth of the drilling hole does not reach the geothermal reservoir, and at the same time, after the drilling of the final hole, the accuracy of the measured temperature varies greatly according to the time when the drilling hole has been stationary, so that direct measurement of the reservoir temperature by on-site drilling is a very expensive and time-consuming work^7,8^. Indirect calculation method, i.e. fluid geothermometer method, which utilizes the relationship between the content of chemical components in underground hot water and gases and the temperature of the reservoir for thermal storage estimation, the basic principle of which is that, after chemical equilibrium is reached between minerals and fluids or different fluids in deep thermal reservoirs, the temperature decreases during the rise of hot water to the surface, but the content of the chemical components remains unchanged, and the temperature in the geothermal reservoirs can therefore be estimated based on the equilibrium temperatures of the chemical reactions^9,10^. Due to their low cost, methods based on geothermometers for predicting reservoir temperatures in geothermal systems have been widely popularized and rapidly developed, among which, there are cation-based methods: Na–K^11–18^, Na–K-Ca^19^, K-Mg^20^; K-Ca^13^; Silica-based methods for geothermometers^21–23^; gas chemistry-based methods for geothermometers, etc.^24–26^. Although some progress has been made in reservoir temperature prediction based on geothermometers, the computational results of the geothermometer method still have large differences when compared with the direct temperature measurement method. In response to the uncertainty of the results, when evaluating the geothermometer results, it is usually necessary to rely on a variety of calculation methods and combine them with the actual characteristics of the site to make a comprehensive judgment, which greatly increases the complexity and workload of the work.

With the popularization of computers and the development of machine learning algorithms, the consideration of bringing hydrogeochemical data into machine learning algorithms for reservoir temperature prediction has become a new approach to explore the prediction of reservoir temperature^27^. Díaz-González et al. (2008) improved three Na–K geothermometer equations using artificial neural networks and linear regression to improve geothermal temperature prediction in geothermal systems^18^; Porkhial et al. (2015) Modeling and prediction of geothermal reservoir temperatures were attempted through a neural network model^28^; Perez-Zarate et al. (2019) employed a three-layer artificial neural network, taking CO_2_, H_2_S, CH_4_, and H_2_ as inputs and bottomhole temperature as output, to perform multivariate analysis on fluid gas composition and predict geothermal reservoir temperature^27^; Tut Haklidir and Haklidir (2020) Reservoir temperatures in western Anatolia, Turkey, were predicted using hydrogeochemical data through linear regression, linear support vector machine and deep neural network methods^29^; Varol Altay et al. (2022) further utilized hydrogeochemical data from different geothermal areas in western Anatolia, Turkey, to propose a hybrid artificial neural network model based on heuristic optimization algorithms for predicting reservoir temperatures^30^. In the same year, Afandi et al. (2022) used Artificial Neural Network (ANN) model to predict probe temperature^31^. Davoodi and Vo Thanh (2023) proposed the LSSVM machine learning model for predicting the residual captive index of carbon dioxide solubility at global geologic sequestration sites, hydrogen uptake values of porous carbon materials, and combined machine learning with optimization to propose the LSSVM-COA model to improve the prediction accuracy while reducing the Associated uncertainties^32–34^; Davoodi and Mehrad (2023) proposed hybrid machine learning for rapid prediction of rheological and filtration properties of water-based drilling fluids, achieving accurate and reliable prediction of filtration properties of drilling fluids and applying hybrid machine learning to assist in prediction of uniaxial compressive strength using drilling variables^35,36^.

Overall, there is a general lack of sufficient training data when scholars adopt machine learning to predict reservoir temperature, while previous studies tend to directly take the collected hydrochemical data as inputs without considering the relationship between different combinations of inputs (hydrochemical data) and outputs (reservoir temperature), which results in the proposed methods having their own scope of applicability without strong generalizability.

The main objective of this paper is to investigate the performance of machine learning models that take into account data characterization and to determine the applicability of using machine learning for reservoir temperature prediction. By searching the literature, 83 sets of hydrogeochemical data and reservoir temperature data were collected, and the characteristics of the dataset were carefully analyzed using normalization, box plots, and mutual correlation analysis; Build a total of four machine learning regression models, Bayesian Ridge Regression, Decision Tree Regressor, eXtremeGradient Boosting (XGBoost) and Light Gradient Boosting Machine (LightGBM); Solve the model accuracy problem caused by a small amount of data through fivefold cross-validation; construct a prediction model considering multiple data combinations through multiple data combination forms. Performance evaluation metrics are used to evaluate the model to demonstrate the method's performance in predicting reservoir temperatures and to identify the optimal algorithms, while various model predictions are compared with traditional geothermometer methods to determine the applicability and accuracy of the prediction models.

## Data preparation and preprocessing

### Data preparation

**Figure 1:**
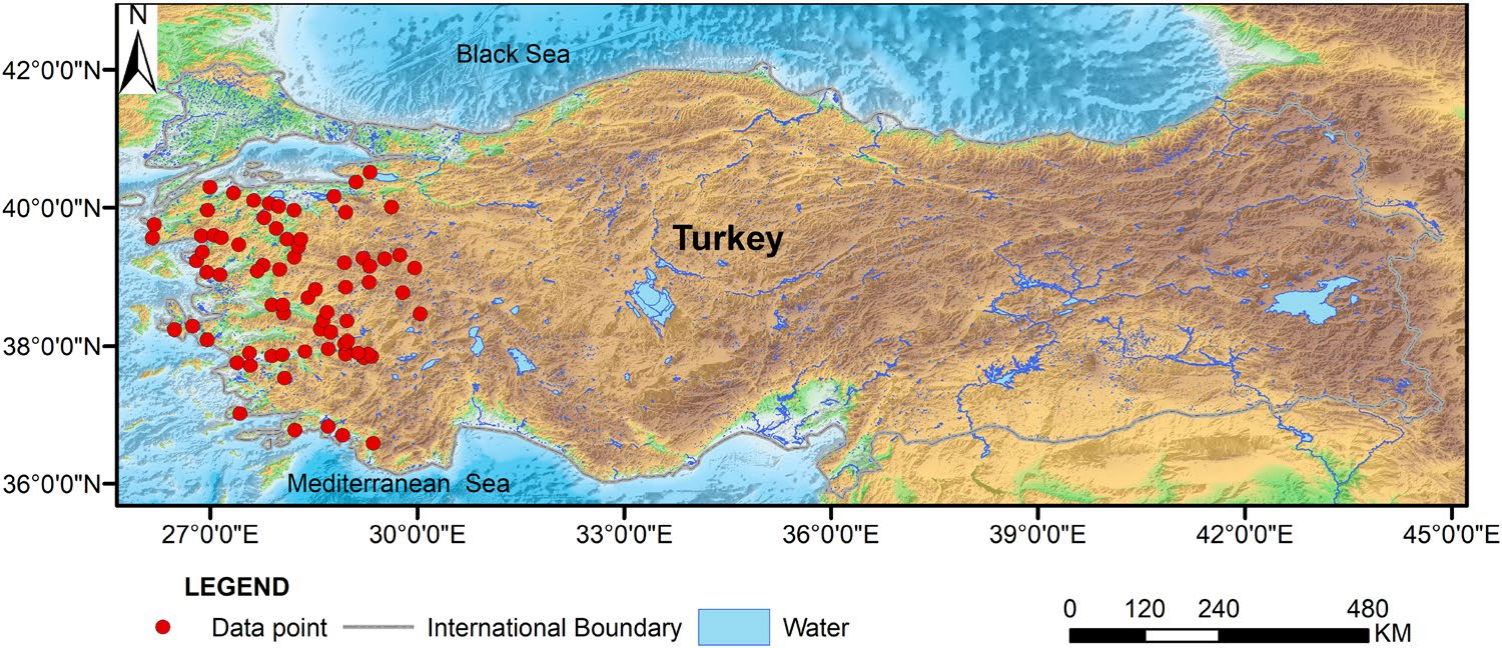
Relief map of western Anatolia, Turkey, showing the location of the study area. Study data from (Tut Haklidir and Haklidir^29^). Base map from Grid Extract (noaa.gov https://www.ncei.noaa.gov/maps/grid-extract/).

The 83 data sets in the paper are from the research dataset of Tut Haklidir and Haklidir (2020), which consists of hydrogeochemical data and reservoir temperatures obtained by different researchers in various regions of Western Anatolia, Turkey (Fig. 1). Each group contains the following data parameters: Pondus Hydrogenii (PH), electric conductivity (EC), K^+^, Na^+^, boron, silicon dioxide (SiO_2_), Cl^-^, temperature (T). PH represents the alkalinity of the water, and can also indicate groundwater mixing, geochemical process information; EC is an important parameter for dissolved solids in geothermal fluids, and has been used to predict reservoir temperatures; K^+^ and Na^+^ are the main cations, and can indicate the interactions between the hot water and rocks; Boron is a trace element, which represents the circulation of groundwater, and a high concentration of boron indicates a high-temperature reservoir in the deep subsurface; SiO_2_ is an important indicator for predicting the geothermal temperature, and the content of silica in geothermal fluids depends on the solubility of quartz in water at different temperatures and pressures. Silica solubility increases with increasing temperature, and dissolved silica in natural water is generally unaffected by other ions, the formation of complexes and volatilization and dissipation, and the rate of precipitation slows down with decreasing temperature, so that the concentration of silica in surface water is a good indicator of the temperature of subsurface thermal reservoirs; Cl^-^ is the main anion, representing the salinity of subsurface hot water; the temperature is the geothermometer-calculated hot spring reservoir temperatures and measured reservoir temperatures from geothermal wells.

Information on data characteristics such as maximum, median, and minimum values are listed in Table 1, where the minimum and maximum values indicate that the dataset attributes vary over a very biao wide range, e.g., conductivity EC in microS/cm ranges from 300 to 10,330, while K^+^ is a concentration (mg/l) ranging from 0.8 to 191. Detailed data are shown in Table 1.

**Table 1:** Summary statistics for the dataset.

	PH	EC (μS/cm)	K^+^ (mg/l)	Na^+^ (mg/l)	Boron (mg/l)	SiO_2_ (mg/l)	Cl^−^ (mg/l)	T (°C)
Max	9.1	10,300	191	1810	38	650	945	245
Median	7.5	2590	50	540	8.6	96	85	131
Min	5.8	300	0.8	2.6	0	11	3	50
Mean	7.55	2976.58	65.29	632.24	10.06	165.89	94.36	144.80
Standard deviation	0.78	1913.41	57.10	513.08	9.91	152.87	352.38	56.21

The accuracy of the data will affect the completeness and accuracy of the research results. The data concentration in deep reservoirs can be affected by the vapor fraction, resulting in higher or lower data concentration^37,38^. Since this study focuses on how to choose the best input parameters and use reasonable machine learning algorithms to obtain more accurate temperatures, the effect of vapor fraction on the data is not considered, and whether the data are from a single well or multiple wells is not considered. In this paper, we will use the normalization method to reduce the data dimensionality and improve the interpretability of the data, as well as to improve the accuracy of the study results through data significance analysis and cross-validation.

### Data preprocessing

The datasets have different scale features (Table 1), which usually have different dimensions, and in most models in machine learning, the different dimensions of the different features cause a large range of values to be calculated. Therefore, the raw data is usually normalized in order to improve the interpretability of the data, reduce noise and redundancy, and ensure better results from the model^39^. In this study, normalization will be used to scale the raw data to between [−1, 1] and then brought into the model for the study. The normalization equation is shown in Eq. (1):

1 $${x}^{*}=\frac{x-{x}_{mean}}{{x}_{max}-{x}_{min}}$$

where: ${x}^{*}$ is the normalized data, ${x}_{mean}$ is the mean of the original data, ${x}_{max}$ and ${x}_{min}$ denote the maximum and minimum values of the original data.

**Figure 2:**
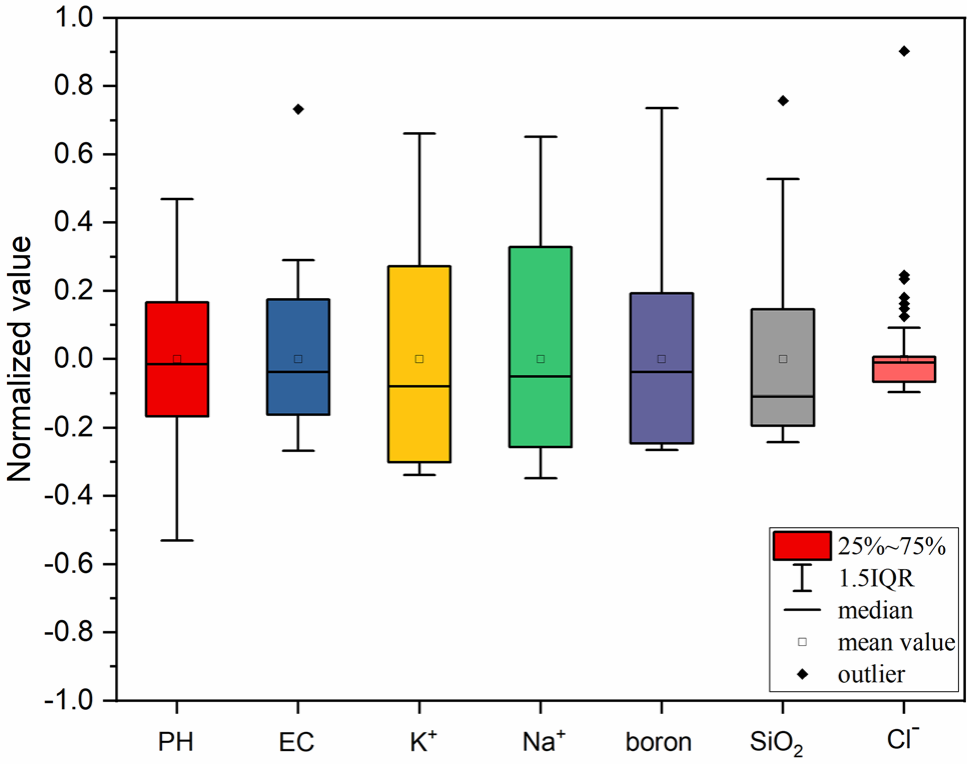
Data feature box diagram.

Box plots in Fig. 2 display the distribution of normalized feature data, where the small boxes represent data means, the horizontal lines across indicate medians, and black diamond-shaped boxes denote data outliers. The figure reveals that there are three sets of data containing outliers, namely electrical conductivity (EC), silicon dioxide (SiO_2_), and Cl^-^. The medians of almost all feature data groups do not align with the centerline of the boxes, implying that the distributions of each data group are asymmetric.

### Data characterization

In machine learning models, the advantages and disadvantages of model training are not only related to the data dimensions, but also the selection of data features is the key to decide whether the model is good or bad. The selection of features not only needs to comprehensively reveal the problem, but also should not increase the computational burden by selecting a large number of features. Reasonable feature selection can simplify the model, speed up the model training speed, make the model have better interpretation, and at the same time can reduce overfitting to improve the generalization ability of the model^40^.

Since the feature data may have different degrees of influence on reservoir temperature, in order to determine the most reasonable combination of features, feature selection was performed before model training using the SelectKBest class, along with the f_regression function which computes numerical correlations, was utilized for feature selection. The f_regression function employs F-tests to calculate coefficients between each feature and the target variable.

**Figure 3:**
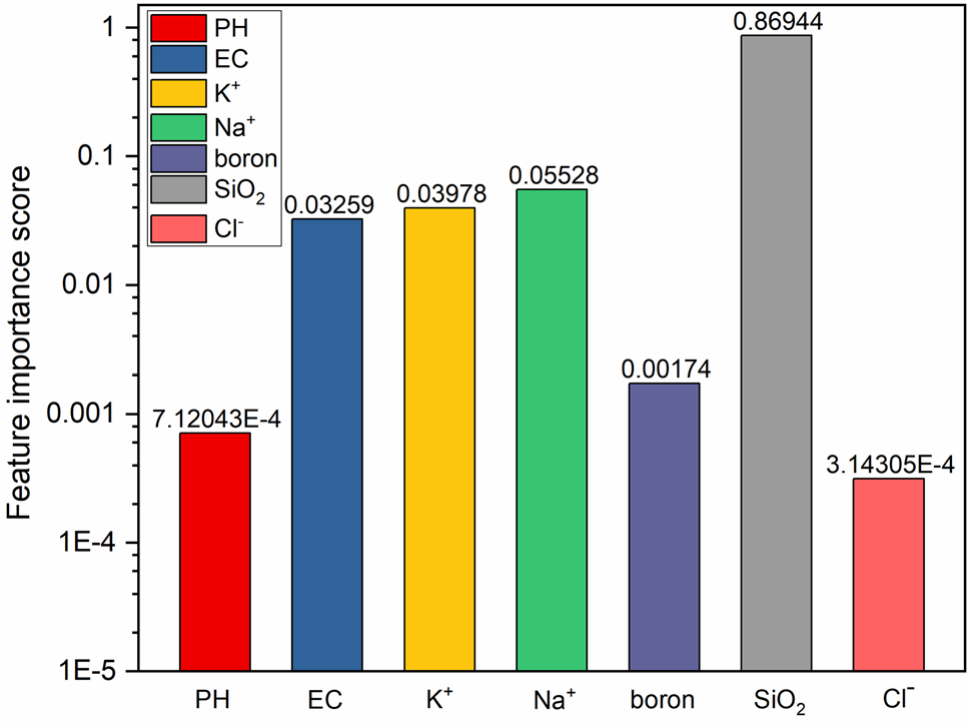
Characteristic importance map.

The intercorrelation equation is shown in Eq. (2):

2 $$F\left(X,Y\right)=\sum_{y\in Y}\sum_{x\in X}p\left(x,y\right){\text{log}}(\frac{p\left(x,y\right)}{p\left(x\right)p\left(y\right)})$$

where: $p\left(x,y\right)$ is the joint probability distribution function of $X$ and $Y$, and $p\left(x\right)$, $p\left(x\right)$ are the marginal probability distribution functions of $X$ and $Y$ respectively. When $X$ and $Y$ are independent random variables, $F\left(X,Y\right)=0$; when X and Y are the same variables, $F\left(X,Y\right)=1$. Therefore, the $F$ value takes the range between [0, 1], the larger the $F$ value, the stronger the correlation between the features and variables. According to the calculation results SiO_2_ correlation is the strongest, its correlation is 0.86944, followed by Na^+^, K^+^ and EC, in order to make the results more intuitive, the vertical coordinate is used logarithmic coordinates, and the importance of the features is plotted (Fig. 3).

### Performance measure

In order to select the best machine learning model for reservoir temperature prediction, it is necessary to measure the predictive ability of the model with the help of evaluation metrics. In the process of model evaluation, it is often necessary to use different indicators for different problems, and among the many evaluation indicators, most of them can only reflect part of the model's performance in a one-sided way, so if they are not used reasonably, not only can they not find out the problems of the model itself, but also they will come to a wrong conclusion. In this paper, we mainly study the regression problem for reservoir temperature prediction, so we choose three indicators, root mean square error (RMSE), mean absolute error (MAE) and decidable coefficient R-Square, for evaluation.

3 $$RMSE=\sqrt{\frac{1}{m}{\sum }_{i=1}^{m}{({\widehat{y}}_{i}-{y}_{i})}^{2}}$$

4 $$MAE=\frac{1}{m}{\sum }_{i=1}^{m}\left|{\widehat{y}}_{i}-{y}_{i}\right|$$

5 $${R}^{2}{=1-\frac{{\sum }_{i=1}{({\widehat{y}}_{i}-{y}_{i})}^{2}}{{\sum }_{i=1}{(\overline{{\widehat{y} }_{i}}-{y}_{i})}^{2}}}_{i}$$

where: root mean square error (RMSE) is the square root of the mean square error. Mean Absolute Error (MAE) is the average of the absolute errors, that is, the average of the errors between the measured value and the true value, which can better reflect the actual situation of the prediction value errors. R-Squared is the value of R-squared.

## Reservoir temperature prediction model

In this paper, based on the Scikit-learn open source algorithm package for machine learning in Python^41^, a total of four machine learning models, namely Bayesian Ridge Regression, Decision Tree Regressor, XGBoost and LightGBM, were used. 80% of the data were used to train the models and 20% of the data were used to validate the models for model building, hyper-parameter optimization and result prediction. The mathematical equations and theoretical methods of the above machine learning can be deeply understood through references^14,42,43^, and only short definitions and applications are given in the paper.

### Bayesian ridge regression

Bayesian Ridge Regression evolved from Bayesian linear regression^44^, which combines the ideas of Ridge Regression and Bayesian statistics. That is, an L2 regularization term (penalty term) is added to the loss function of Bayesian Ridge Regression to control the complexity of the model, and the core idea is to introduce a prior distribution (usually Gaussian) into the loss function to describe the uncertainty of the parameters, and then estimate the model parameters by Maximum A Posteriori Estimation (MAP), which is equivalent to minimizing the loss function while considering the prior distribution of the parameters.

6 $$p\left(w|\lambda \right)=N\left(w|\alpha ,{\lambda }^{-1}{I}_{p}\right)$$

where ${I}_{p}$ is the order unit square, $N$ is the Gaussian distribution, $\alpha$ is the hyperparameter mean, and $\lambda$ is the standard deviation.

The regularization term in the algorithm can enhance the robustness of the model, and it is not easy to overfit when the sample size of this study is small. At the same time, the algorithm provides a probabilistic framework, allowing the uncertainty of the prediction to be quantified, taking into account the needs of data fitting and the stability of parameters, and has the advantages of robustness and high precision. However, assuming that there is a linear relationship between the predictor variable and the response variable, it may not be able to capture the complex nonlinear pattern in the data, and the model has the problems of large amount of data calculation and long calculation time.

### Decision tree regression

Decision tree regression is a regression analysis method based on decision trees^45^, based on the powerful algorithms of decision trees, which are able to fit complex datasets better even when faced with some complex problems. A decision tree is a tree structure in which each internal node represents a test of a feature attribute, each branch represents a test output, and each leaf node represents a predicted value. Starting from the root node, based on the test results of each internal node, the samples are assigned to different child nodes until the leaf node is reached, the average target value of the training samples in the leaf node is the predicted value, the regression tree predicts at each node for a specific value, and when splitting the training set the goal is to find a split that minimizes the MSE.

Each regression tree corresponds to a division of the input space and an output value on the redivision cell. Assuming that the input space is partitioned into M cells $R1,R2,... , Rm$, and there is a fixed output value $Cm$ on each cell $Rm$, the regression tree model can be expressed as:

7 $$f\left(x\right)={\sum }_{m=1}^{M}{c}_{m}I(x\in {R}_{m})$$

In this study, the data contains nonlinear features, and the decision tree can flexibly adapt to this complex relationship. Simultaneous decision trees are able to efficiently process multiple features, including multiple variables involved in the prediction, such as PH, conductivity, ion concentration, etc. But decision trees can perform poorly when faced with highly complex relationships.

### XGBoost

eXtremeGradient Boosting (XGBoost) is an integrated learning algorithm based on Gradient Boosting Tree^46,47^, and the basic idea is to build a more powerful predictive model by combining multiple weak learners (usually decision trees). The model is iterated and optimized with each round of gradient boosting to provide superior predictive performance. The optimization objective function of the XGBoost model is:

8 $${\sum }_{i=1}^{n}[{g}_{i}{f}_{t}\left({x}_{i}\right)+\frac{1}{2}{h}_{i}{f}_{t}^{2}\left({x}_{i}\right)]+\Omega ({f}_{t})$$

The core of XGBoost is the gradient boosting tree algorithm, which continuously adds different trees to the model and grows the tree model through feature splitting, each time a tree is added it is equivalent to learning a new function, which gradually improves the performance of the model by training a series of weak learners iteratively, where each new weak learner corrects the prediction error of the previous round of weak learners. Meanwhile, XGBoost supports parallel processing, which enables it to effectively utilize multi-core processors to accelerate the model training process and improve the training efficiency. XGBoost uses L1 and L2 regularization, similar to ridge regression and LASSO, to control the complexity of the model, to prevent overfitting, and to improve the generalization ability of the model. Since the model contains multiple tunable parameters, careful parameter tuning is required and too many parameter choices can lead to overfitting. Due to the sensitivity to outliers, it is often necessary to handle outliers in the preprocessing stage to avoid their negative impact on the model.

### LightGBM

Light Gradient Boosting Machine (LightGBM) is a high-performance gradient boosting tree algorithm^48,49^ similar to XGBoost. LightGBM is optimized in terms of data structure and algorithms to make it perform well in large-scale data and high-dimensional feature cases to perform well.

.LightGBM is suitable for a wide range of classification and regression problems, but proper tuning of the hyper-parameters is required to achieve optimal performance. The algorithm is based on a histogram-based learning approach and is applicable to multiple geological and chemical variables that may be involved in reservoir temperature prediction. A tree-based learning algorithm is employed to support parallel computing with high training and prediction speeds. The ability to flexibly capture nonlinear relationships in reservoir temperature data provides more accurate predictions. Meanwhile, the algorithm has a relatively low memory footprint and is suitable for use in resource-limited environments. However, it is more sensitive to outliers, which need to be dealt with in the preprocessing stage to avoid their negative impact on the model.

### Hyperparameters tuning

**Figure 4:**
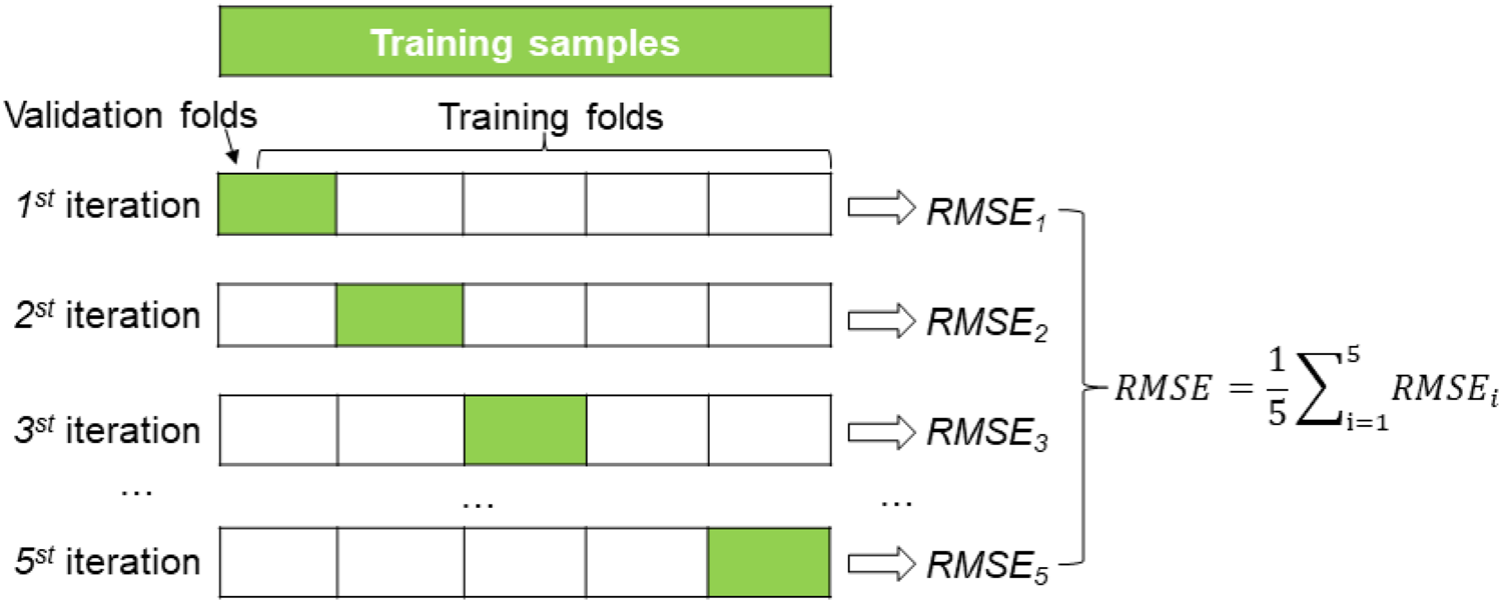
5-fold cross-validation.

Previous studies usually directly divide the data into training set and testing set proportionally, when the data set is not large enough, different division methods, different models are obtained, and when the division method is not good enough, it is difficult to select a good model and parameters, cross-validation is an effective way to solve this problem^50^. N-fold cross-validation is performed by randomly dividing the data into n groups, with n − 1 group for training and 1 group for validation, each subset of the group is used as validation data, loop n times to train n model results, and average the results of all the groups to produce the individual accuracy of the model as the final result (Fig. 4).This method can effectively prevent model overfitting, and at the same time, it is better able to find more appropriate model parameters.

For the small dataset problem in this paper, GridSearchCV in sklearn is used to search for the optimal parameters and re-fit the model.GridSearchCV has two functions, grid search and cross-validation, which ensures that the parameter with the highest accuracy can be found within the specified parameter range, and auto-tuning, so that as long as the parameter is inputted, the optimal result and the parameters will be given.

## Results and discussion

### Comparison of different machine learning algorithms

The learning and training of four algorithms are implemented based on the sklearn machine learning package in the python open source library, and the hyperparameters of each algorithm are set as follows (Table 2), and the optimal parameters are selected by the GridSearchCV search in "Hyperparameters tuning".

**Table 2:** Structure parameters.

BayesianRidge Regression	lpha_1	(−8, 0, 10)
	lpha_2	(−8, 0, 10)
	ambda_1	(−8, 0, 10)
	ambda_4	(−8, 0, 10]
Decision Tree Regression	best_tree	[3, 5, 6, 7, 8, 9]
XGBoost	max_depth	[3, 5, 7]
	learning_rate	[0.01, 0.05, 0.1]
	n_estimators	[100, 500, 1000]
	subsample	[0.5, 0.7, 1.0]
	colsample_bytree	[0.5, 0.7, 1.0]
	reg_alpha	[0.01, 0.1, 1.0]
	reg_lambda	[0.01, 0.1, 1.0]
LightGBM	num_leaves	[20, 30, 40]
	learning_rate	[0.01, 0.05, 0.1]
	max_depth	[4, 5, 6]
	min_child_weight	[0.1, 1, 5]

#### BayesianRidge regression

Four control parameters, lpha_1 controls the normal prior, lpha_2 controls the observation error, ambda_1 controls the strength of all regression coefficients gradually approaching 0, and ambda_4 controls the strength of all regression coefficients gradually approaching a common value. The optimized values of the control parameters were obtained by applying a grid search. Table 2 shows the range of control parameter values chosen to obtain the optimal parameters, which were obtained from the search: lpha_1: 1.0, lpha_2: 1e−08, ambda_1: 1e−08, ambda_2: 1.

#### Decision tree regression

Decision tree regression mainly controlled by the maximum depth of the tree, in order to prevent overfitting in Table 2 set up for obtaining the optimal parameters of the control range, through the grid search to obtain the optimal decision tree model depth of 6.

#### XGBoost

XGBoost consists of seven control parameters, max_depth controls the maximum depth of the tree; learning_rate indicates the learning rate, which controls the iteration rate and prevents overfitting; n_estimators indicates the number of integrated weak estimators, the larger the n estimators are, the stronger the learning ability of the model is, and the easier the model is to overfitting; subsample controls the proportion of sampling from the sample; colsample_bytree controls the proportion of all features randomly sampled when constructing each tree; reg_alpha:L1 regularization coefficient; reg_lambda: :L2 regularization coefficient. Table 2 shows the range of control parameter values chosen to obtain the optimal parameters. The optimal parameters obtained from the search are: max_depth: 3, learning_rate: 0.1, n_estimators: 1000, subsample: 0.7, colsample_bytree: 1.0, reg_alpha. 0.01, reg_lambda: 1.0.

#### LightGBM

LightGBM controlled by four parameters, num_leaves is the number of leaf nodes on a tree, and max_depth to control the shape of the tree, the parameter has a great impact on the performance of the model, need to focus on regulating the parameters. learning_rate indicates the learning rate, choose a relatively small learning rate can obtain stable and better model performance, max_depth controls the maximum depth of the tree, too large a value of overfitting will be more serious, min_child_weight is the sum of all the samples in the smallest child node, the parameter is too large the model will be underfitting, too small will lead to overfitting, need to be adjusted according to the data. The model will be underfitted if the parameter is too large and overfitted if it is too small. The optimal parameters obtained through grid search are: num_leaves: 20, learning_rate: 0.05, max_depth: 4, min_child_weight: 0.1.

**Figure 5:**
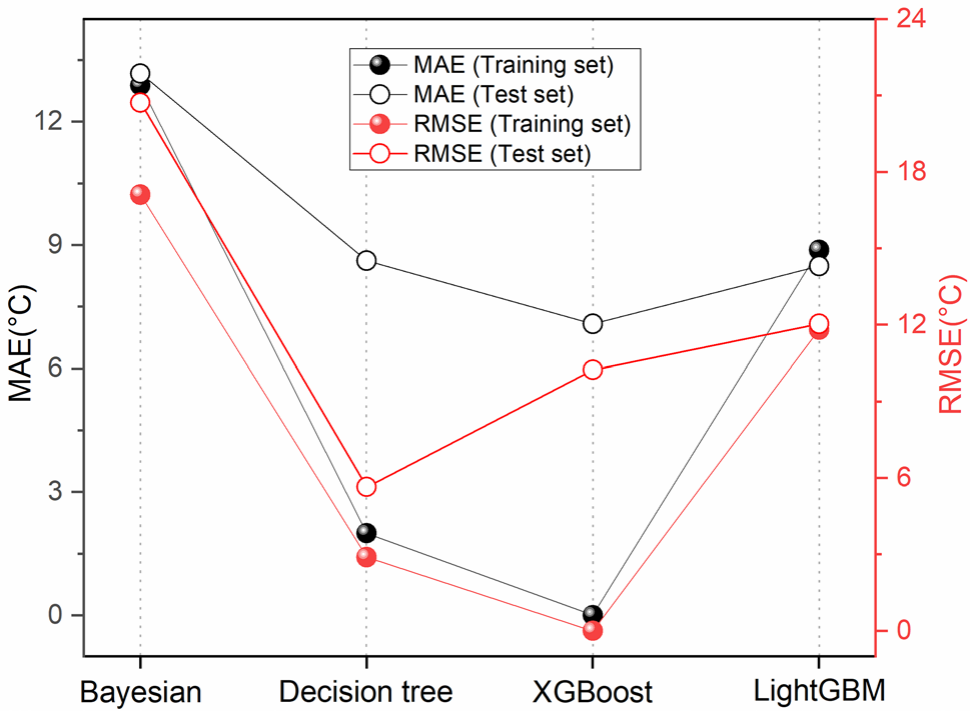
MAE and RMSE for the training and test sets of the 4 models (the solid center of the figure shows the training set and the hollow center shows the results of the test set; MAE is shown in black and RMSE is shown in red).

The prediction performance of the four machine learning algorithms was analyzed by optimal parameter selection, and Fig. 5 shows the prediction errors of the training dataset and the test dataset. From the figure, it can be concluded that the prediction error of the test dataset is basically similar to that of the training dataset. Among the four methods, the MAE of the XGBoost training dataset and the test dataset are 0.002 and 7.08, and the RMSE is 0.003 and 10.24, indicating that the algorithms have relatively low generalization. The LightGBM training dataset and the test dataset have the highest MAE and RMSE errors are the smallest and the generalizability is the highest. all four algorithms have good MAE and RMSE, indicating that the above machine learning algorithms can be used to predict reservoir temperature.

**Figure 6:**
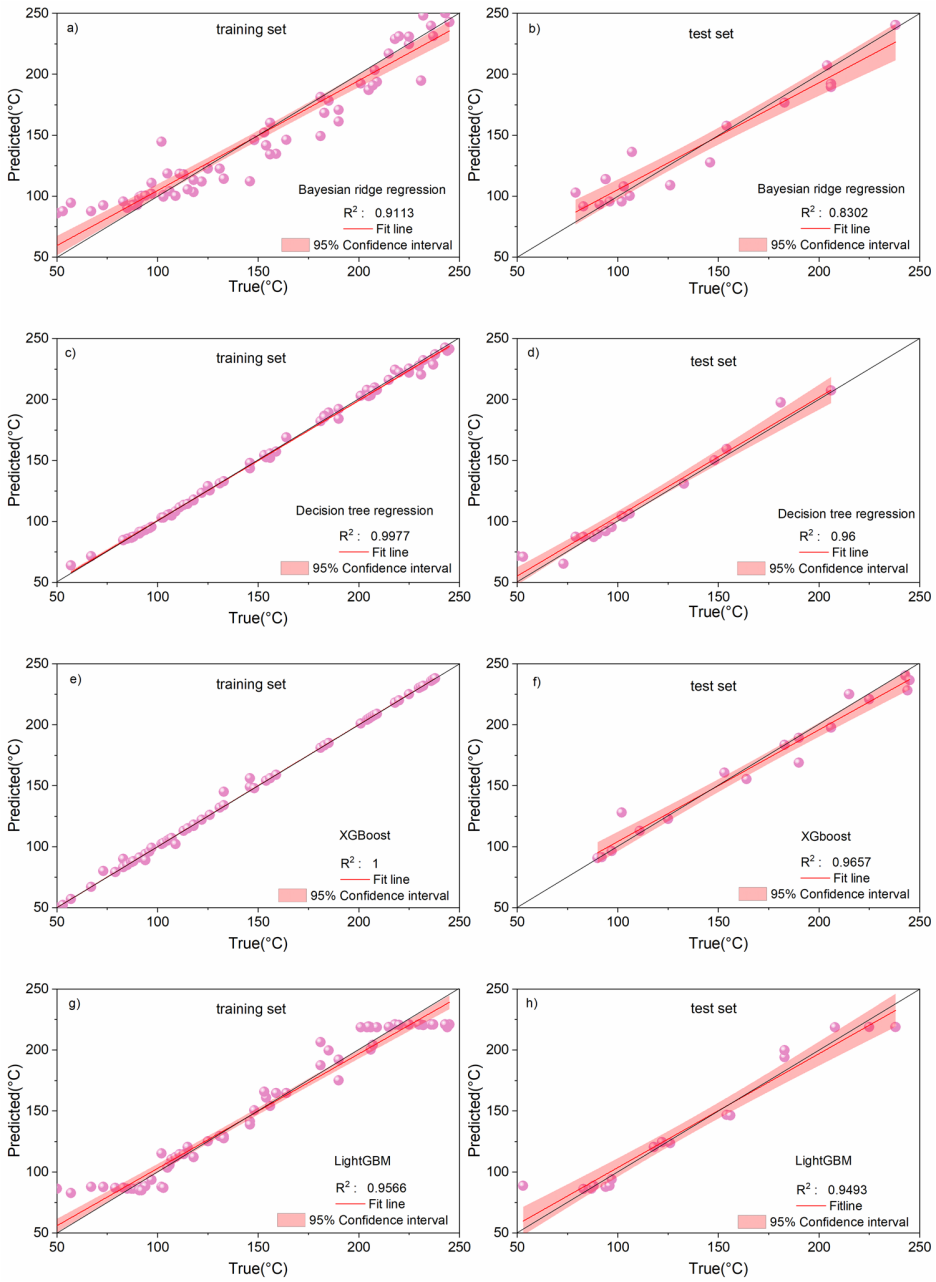
Prediction results and R^2^ for the training and test sets of the 4 models.

The prediction results of the training set and test set were plotted (Fig. 6), all the models had good prediction results in the training set. Migrating the trained models to the test set, the results showed that, the R^2^ of Bayesian Ridge Regression Algorithm = 0.8302, Decision Tree Algorithm R^2^ = 0.96, XGBoost Algorithm R^2^ = 0.9657 and LightGBM Algorithm R^2^ = 0.9493, Except for the Bayesian ridge regression algorithm, the predicted values of the three tree-based algorithms match well with the true values in the test set (all of them reach more than 94%). This shows that the tree-based machine learning algorithms can accurately predict the underground reservoir temperature. After the comprehensive evaluation of MAE, RMSE and R^2^ indexes, it can be seen that the machine learning algorithm using XGBoost has the best accuracy in predicting the results, and LightGBM and decision tree algorithms are the second best.

### Comparison of different feature combinations

In this section, based on the correlation of features (Fig. 3) and combining the significance of different features, different forms of feature combinations are constructed and listed in Table 3, which are compared with the prediction performance of F-4 to explore the effect of feature selection on the temperature prediction performance.

**Table 3:** Four different feature combinations.

Feature Combinations	PH	EC (μS/cm)	K^+^ (mg/l)	Na^+^ (mg/l)	Boron (mg/l)	SiO_2_ (mg/l)	Cl− (mg/l)
F-1		√	√	√		√	
F-2		√	√	√	√	√	
F-3	√	√	√	√	√	√	
F-4	√	√	√	√	√	√	√

**Figure 7:**
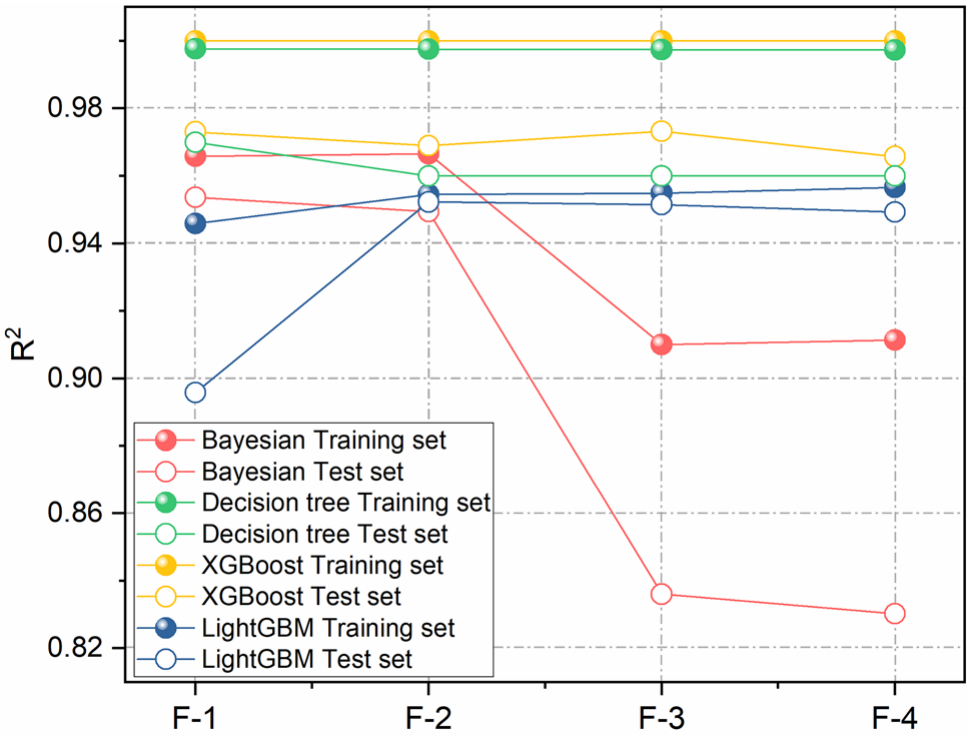
R^2^ of different algorithms using different feature combinations.

Four algorithms were used to train the model for each of the above forms of feature combinations, and the prediction results of the training and test sets were plotted (Fig. 7). The results show that Bayesian Ridge regression has a better predictive ability of the model when choosing reasonable input parameters (F-1, F-2), and the predictive effect of the model decreases with the input of lower influencing factors (Fig. 7, red F-3, F-4); Decision Tree regression and XGBoost model have a small difference in the prediction error of the reservoir temperature under the conditions of different feature combinations (green and yellow dotted lines); LightGBM model has higher predictive ability of the model at F-2 and F-3, and the predictive effect of the model decreases at F-1 and F-4 (blue dotted line).

The test results of the four algorithms with the four combination forms are further compared (Fig. 7 and Table 4). The results show that the model accuracies of different algorithms with different feature combinations range from 0.8302 to 0.9732, and XGBoost performs the best with an average accuracy of 0.9703, followed by Decision Tree algorithm with an average accuracy of 0.9625, and LightGBM with an average accuracy of 0.9373. Among them, XGBoost and Decision Tree algorithms do not depend on the selection of features, and XGBoost does not depend on the selection of features with different feature combinations. XGBoost and Decision Tree algorithms are not strongly dependent on the selection of features, and the accuracy of XGBoost is in the range of 0.9657 to 0.9732 for different combinations of features; Decision Tree algorithm is the next best, and its accuracy is in the range of 0.96 to 0.97 for different combinations of features.The average value of the accuracy of Bayesian Ridge The accuracy of Bayesian Ridge Regression and LightGBM algorithms is unstable and sensitive to the selection of features, Bayesian Ridge Regression algorithm has the largest fluctuation in accuracy, with a minimum of 0.8302 when using the feature combination F-4, and a maximum of 0.9537 when using the feature combination F-1; LightGBM algorithm has a maximum of 0.9537 when using the feature combination F-1. The accuracy of LightGBM algorithm is slightly lower than 0.9 when only feature combination F-1 is used, and the average accuracy of different algorithms is maximum 0.9577 when feature combination F-2 is used, followed by feature combination F-1 (the average accuracy of the algorithms is 0.9482), and the average accuracy of the algorithms is minimum 0.9263 when feature combination F-4 is used.

**Table 4:** R^2^ of different algorithms using different feature combinations.

Feature combinations	Bayesian Ridge Regression	Decision Tree Regressor	XGBoost	LightGBM	Mean
F-1	0.9537	0.9700	0.9731	0.8958	0.9482
F-2	0.9494	0.9600	0.9690	0.9523	0.9577
F-3	0.8360	0.96	0.9732	0.9516	0.9302
F-4	0.8302	0.96	0.9657	0.9493	0.9263
Mean	0.8923	0.9625	0.9703	0.9373	

By comparing the prediction performance of the four modeling algorithms with the F-4 combination, it is shown that a reasonable selection of input features can improve the prediction results of the model.

**Figure 8:**
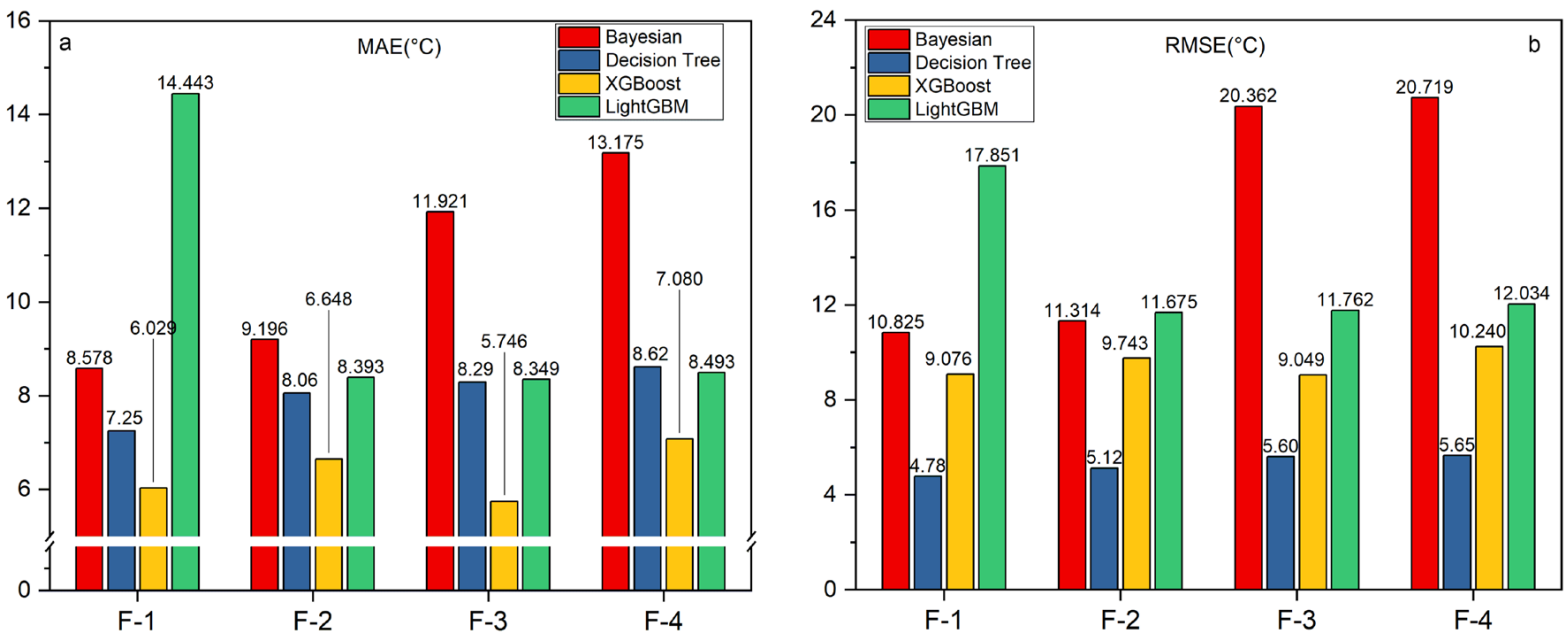
MAE and RMSE of different algorithms using different combinations of features.

Furthermore, the evaluation results of different algorithms using various feature combinations based on the MAE and RMSE metrics are presented in Table 5 and Fig. 8. The results indicate that, for different algorithms using different feature combinations, the Mean Absolute Error (MAE) ranges from 6.0287 to 14.4431, and the Root Mean Squared Error (RMSE) ranges from 4.78 to 20.7185. XGBoost and Decision Tree algorithms exhibit the smallest prediction errors. XGBoost achieves an MAE ranging from 6.0287 to 7.08 across different feature combinations, followed by the Decision Tree algorithm with an MAE range of 7.25 to 8.62. However, Bayesian Ridge Regression and LightGBM algorithms exhibit instability and sensitivity to feature selection. When using only feature combination F-1, the LightGBM algorithm shows larger MAE and RMSE values. The Bayesian Ridge Regression algorithm demonstrates the greatest fluctuation in error, with RMSE exceeding 20 when using feature combinations F-3 and F-4. With feature combination F-2, the algorithms exhibit the smallest average errors, with an MAE of 7.3392 and RMSE of 10.1981. Next is feature combination F-3, with an average MAE of 7.9094 and RMSE of 12.3658. Feature combination F-4 results in the largest average errors for the algorithms, with an MAE of 8.5995 and RMSE of 12.9033.

**Table 5:** MAE and RMSE of different algorithms using different feature combinations.

Feature combinations	Criterion	Bayesian ridge regression	Decision tree regression	XGBoost	LightGBM	Mean
F-1	MAE	8.5781	7.25	6.0287	14.4431	8.4575
	RMSE	10.8248	4.78	9.0757	17.8509	11.2503
F-2	MAE	9.1957	8.06	6.6483	8.3928	7.3392
	RMSE	11.3144	5.12	9.7433	11.6747	10.1981
F-3	MAE	11.9209	8.29	5.74627	8.3489	7.9040
	RMSE	20.3617	5.6	9.0491	11.7625	12.3658
F-4	MAE	13.1750	8.62	7.08	8.4929	8.5995
	RMSE	20.7185	5.65	10.2402	12.0344	12.9033

**Figure 9:**
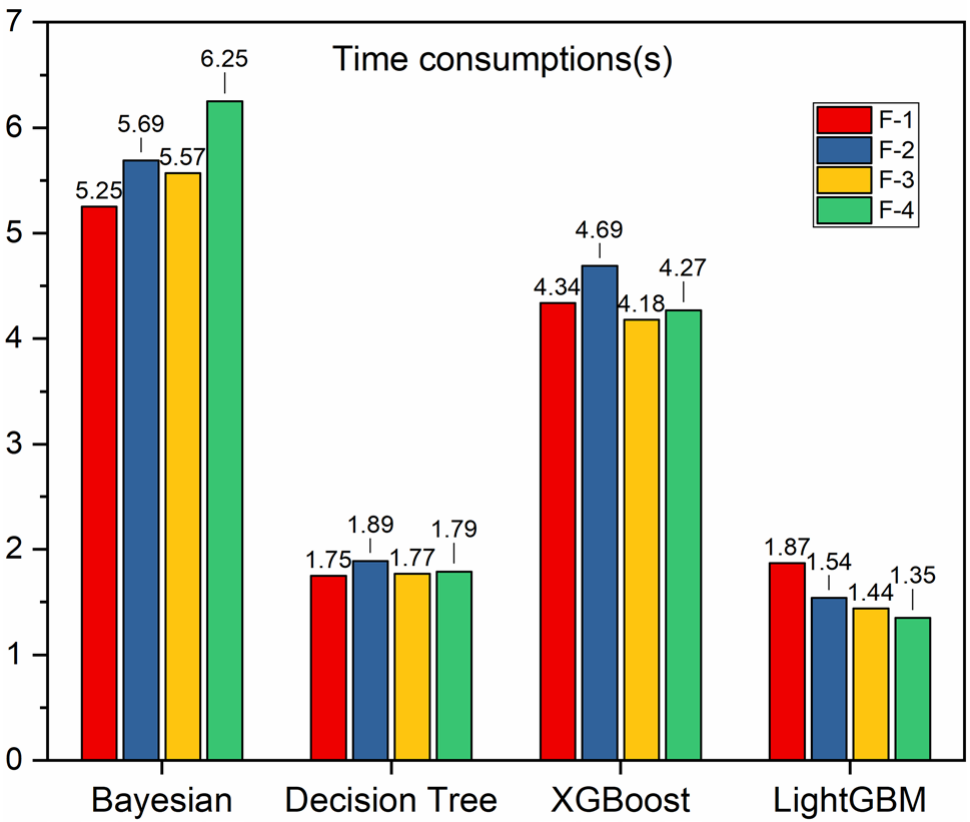
Time consumptions for learning curves of machine learning models.

We further calculated the running time of each model under various combinations (Fig. 9), which reflects the computational performance of each model by recording the execution time of each model's learning curve, and the sum of the validation time for the validation sample size. Usually, an optimal model requires less running time, while a bad model will be very time-consuming, as can be seen in Fig. 9, the running efficiencies are LightGBM, Decision Tree Regression, XGBoost, and Bayesian Ridge Regression in descending order.

Taking into consideration the evaluation results of the four metrics mentioned above, the optimal combination of reservoir temperature prediction is as follows: when the feature combination F-3 is adopted and XGBoost algorithm is selected, the model error is minimum and the accuracy is maximum of 0.9732.

### Comparison with traditional geothermometer methods

**Figure 10:**
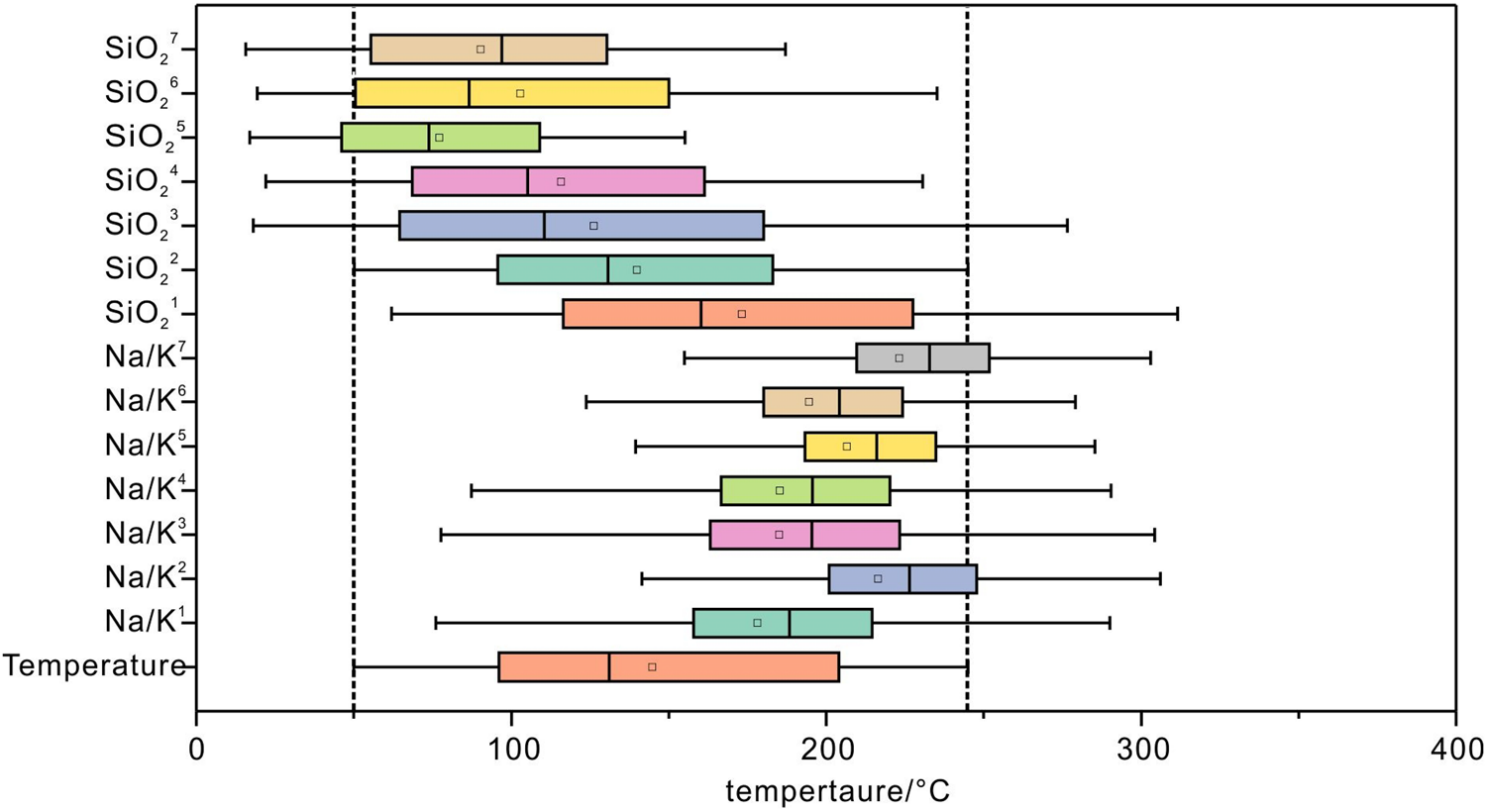
Comparison of predicted reservoir temperature with measured temperature based on chemical geothermometer.

Based on the sodium–potassium cation and SiO_2_-based geothermometers (Suppl. Appendix B), a comparison between the predicted reservoir temperatures using the geochemical geothermometer formula and the measured temperatures is presented (Fig. 10). and The sodium–potassium geothermal temperature scale formula is based on cation-exchange reactions and does not apply to hot water where mixing of hot water of different origins occurs, nor does it apply to acidic water with a pH much less than 7^11–13^. The silica geothermometer method is based on the solubility of silica minerals and is applicable in the interval of 20 to 250°C^51^. Therefore, only the alkaline (pH greater than 7) dataset was selected for the calculations when sodium–potassium geothermal temperature scaling method was used.

The results show that the Na/K^1^ geothermometer has good prediction results mainly for reservoirs above 150 °C and is not applicable to this dataset; the results of the geothermometers by Na/K^2^, Na/K^3^, Na/K^4^, Na/K^5^ Na/K^6^ and Na/K^7^ are much larger than the actual temperatures, which are not in accordance with the actual situation. For the SiO_2_ ground thermometer, the estimated temperature of SiO_2_^1^ is slightly larger than the actual temperature; the estimated temperatures of SiO_2_^4^, SiO_2_^5^, SiO_2_^6^, and SiO_2_^7^ are lower than the actual temperature; the estimated temperatures of SiO_2_^2^ and SiO_2_^3^ are close to the actual temperature and provide reasonable predictions for the geothermal field. The SiO_2_^2^ and SiO_2_^3^ methods yield Mean Absolute Errors (MAE) of 30.80 and 51.16, respectively, and Root Mean Square Errors (RMSE) of 57.56 and 71.65, respectively.

The SiO_2_^2^ and SiO_2_^3^ geothermometer estimation results are compared with the machine learning based reservoir temperature prediction. All the prediction results based on machine learning are superior to the SiO_2_^2^ and SiO_2_^3^ geothermometer method. The difference in the error distribution of the two types of geothermometers is large, which indicates that the machine learning algorithms have a certain degree of superiority.

### Generalizability analysis of the model

In the most ideal case, we expect the model to perform without substantial performance bias when applied to different datasets. That is, a model with good generalization is able to successfully apply the patterns or laws learned during training to new and different datasets, rather than just performing well on the training data. In this section, we validate the generalizability of the model by applying data from previous published results [Shadfar Davoodi, Hung Vo Thanh (2023), Shadfar Davoodi, Mohammad Mehrad (2023)], brought into our trained model for prediction. Table 6 compares the RMSE, MAE and R^2^ values achieved using the modeling algorithms of this study for predictions presented in published studies. The results in Table 6 show that placing new data into the model is still able to make good predictions and the most accurate predictions are for the XGBoost model of this study with RMSE ,MAE and R^2^ of 0..328, 0.228 and 0.997 respectively.

**Table 6:** Model performance in previously studied data.

ML model	RMSE	MAE	R^2^	Data sources
Bayesian ridge regression	17.105	12.884	0.911	Davoodi and Vo Thanh (2023)^34^
Decision tree regression	12..719	9.917	0.927	
XGBoost	10.240	7.081	0.968	
LightGBM	11.805	8.876	0.956	
Bayesian ridge regression	8.167	6.495	0.827	Davoodi and Mehrad (2023)^36^
Decision tree regression	3.467	2.161	0962	
XGBoost	0.328	0.228	0.997	
LightGBM	12.596	8.565	0.882	

## Conclusions

In this paper, reservoir prediction models with different machine learning algorithms were trained using the same dataset based on the geothermal dataset of western Turkey, and the prediction performance of Bayesian Ridge Regression, Decision Tree Regression, XGBoost, and LightGBM was compared, and the effect of different feature combinations on the prediction performance of reservoir temperature was investigated, and the results were compared with those of the estimates from traditional geothermometers. Based on the above studies, the main findings are as follows:

- Without considering the data features, among the four algorithms, the machine learning algorithm of XGBoost has the best accuracy of R^2^ = 0.9657, followed by LightGBM and Decision Tree algorithms.
- By comparing the predictive results of different feature combinations, a reasonable selection of input features can improve the prediction results and prediction efficiency of the model.
- When the optimal combination for reservoir temperature prediction is feature combination F-3 and the XGBoost algorithm is chosen, the model error is minimized, achieving the highest accuracy of 0.9732.
- The prediction accuracy and stability of the machine learning method are obviously better than that of the traditional geothermometer method, and the results of this study can help the application of machine learning in reservoir temperature prediction and extend it to the related fields of engineering geology.

### Outlook

In the future, the effect of deep reservoir data characteristics on reservoir temperature will be further explored, and the effect of steam fractions on element concentration will be considered in the model and whether the data is from a single well or multiple Wells.

### Supplementary Information

Supplementary Information 1.

Supplementary Information 2.

## Data Availability

The data that support the findings of this study are available from the corresponding author upon reasonable request, and the underlying data are given in the Appendix of this paper.
