# Supplementary material for: Reservoir temperature prediction based on characterization of water chemistry data—case study of western Anatolia, Turkey
Source: Sci Rep. 2024 May 6;14:10339. doi: 10.1038/s41598-024-59409-5 (PMC11074159; doi:10.1038/s41598-024-59409-5)
Supplement: Supplementary file 1 — Supplementary Information 1. [file 41598_2024_59409_MOESM1_ESM.docx]

# Appendix A. Raw data

| Labels | Temperature  (°C) | PH | EC  (microS/cm) | K^+^  (mg/l) | Na^+^  (mg/l) | Boron  (mg/l) | SiO_2_  (mg/l) | Cl^-^  (mg/l) |
| --- | --- | --- | --- | --- | --- | --- | --- | --- |
| 1 | 103 | 8.8 | 1160 | 4 | 227 | 1.4 | 50 | 62 |
| 2 | 146 | 8.5 | 2000 | 21 | 430 | 7.1 | 132 | 265 |
| 3 | 107 | 9 | 1440 | 4 | 230 | 2 | 56 | 945 |
| 4 | 67 | 7.2 | 960 | 4 | 40 | 0.15 | 19 | 33 |
| 5 | 88 | 7.5 | 580 | 5 | 23 | 0.2 | 34 | 13 |
| 6 | 91 | 6.9 | 1349 | 18 | 198 | 1.8 | 38 | 8 |
| 7 | 87 | 7.1 | 558 | 5 | 36 | 0 | 33 | 7 |
| 8 | 131 | 6.8 | 1900 | 72 | 440 | 1.7 | 96 | 62 |
| 9 | 126 | 7 | 1215 | 15 | 168 | 1.9 | 87 | 9 |
| 10 | 87 | 7.1 | 582 | 4 | 25 | 0.2 | 34 | 3 |
| 11 | 90 | 7 | 557 | 4 | 23 | 0.3 | 36 | 3 |
| 12 | 88 | 7.1 | 568 | 4 | 24 | 0.2 | 34 | 3 |
| 13 | 50 | 6.9 | 400 | 2 | 20 | 0 | 11 | 5 |
| 14 | 91 | 7.4 | 490 | 3 | 24 | 0 | 37 | 9 |
| 15 | 118 | 7.1 | 1500 | 7 | 220 | 0 | 72 | 29 |
| 16 | 85 | 6.7 | 1250 | 3 | 15 | 0 | 32 | 10 |
| 17 | 102 | 7.2 | 800 | 2 | 134 | 0.2 | 49 | 27 |
| 18 | 91 | 8.3 | 1700 | 5 | 320 | 1.3 | 38 | 81 |
| 19 | 73 | 8.1 | 3300 | 24 | 550 | 5 | 23 | 234 |
| 20 | 57 | 7.4 | 1900 | 15 | 590 | 0 | 14 | 93 |
| 21 | 181 | 7.9 | 3700 | 64 | 530 | 0 | 210 | 90 |
| 22 | 185 | 6.9 | 4870 | 94 | 865 | 17 | 270 | 95 |
| 23 | 154 | 7.9 | 3680 | 114 | 870 | 11 | 155 | 85 |
| 24 | 183 | 6.9 | 300 | 86 | 686 | 16 | 259 | 78 |
| 25 | 154 | 6.5 | 3980 | 75 | 586 | 10 | 154 | 73 |
| 26 | 159 | 6.4 | 4730 | 50 | 535 | 9 | 170 | 134 |
| 27 | 146 | 6.2 | 3850 | 65 | 583 | 8.6 | 132 | 145 |
| 28 | 206 | 6.5 | 4401 | 73 | 564 | 13 | 375 | 87 |
| 29 | 133 | 5.8 | 1850 | 42 | 260 | 3.2 | 100 | 34 |
| 30 | 156 | 6.3 | 2700 | 56 | 540 | 7.5 | 160 | 90 |
| 31 | 106 | 6.3 | 2880 | 21 | 120 | 2.2 | 55 | 29 |
| 32 | 97 | 7.7 | 2500 | 22 | 130 | 2.7 | 43 | 34 |
| 33 | 106 | 6.2 | 2340 | 48 | 38 | 0.9 | 55 | 15 |
| 34 | 148 | 6.2 | 3120 | 113 | 860 | 9.2 | 136 | 93 |
| 35 | 133 | 8.1 | 1388 | 41 | 239 | 8.5 | 100 | 142 |
| 36 | 102 | 7.6 | 10330 | 155 | 1810 | 9.4 | 49 | 315 |
| 37 | 125 | 9.1 | 2590 | 33 | 575 | 9 | 85 | 98 |
| 38 | 122 | 8.7 | 2360 | 11 | 310 | 0.4 | 80 | 43 |
| 39 | 103 | 8.9 | 3600 | 5 | 375 | 1 | 51 | 37 |
| 40 | 109 | 6.7 | 970 | 9 | 240 | 0.7 | 58 | 18 |
| 41 | 111 | 8.4 | 1650 | 46 | 520 | 8.9 | 62 | 71 |
| 42 | 113 | 8 | 1755 | 48 | 560 | 7.4 | 64 | 71 |
| 43 | 94 | 8.4 | 1770 | 48 | 540 | 8.7 | 40 | 76 |
| 44 | 105 | 7 | 2260 | 65 | 503 | 13.6 | 53 | 62 |
| 45 | 118 | 7.6 | 907 | 24 | 198 | 16 | 72 | 37 |
| 46 | 79 | 8 | 1558 | 13 | 240 | 17.4 | 27 | 58 |
| 47 | 153 | 7.2 | 3800 | 106 | 1020 | 11 | 150 | 127 |
| 48 | 115 | 7.9 | 1400 | 19 | 148 | 0.7 | 68 | 38 |
| 49 | 244 | 8.5 | 5480 | 191 | 1464 | 30 | 460 | 144 |
| 50 | 181 | 8.2 | 5090 | 99 | 1332 | 19 | 261 | 212 |
| 51 | 232 | 7.9 | 5770 | 140 | 1513 | 33 | 492 | 212 |
| 52 | 218 | 7.3 | 5370 | 140 | 1389 | 29 | 444 | 248 |
| 53 | 236 | 7.5 | 5890 | 140 | 1508 | 31 | 503 | 326 |
| 54 | 206 | 8.7 | 4790 | 118 | 1225 | 19 | 257 | 99 |
| 55 | 209 | 8.6 | 4840 | 125 | 1240 | 19 | 258 | 99 |
| 56 | 204 | 8.8 | 4950 | 128 | 1273 | 20 | 310 | 101 |
| 57 | 205 | 8.1 | 4490 | 108 | 1149 | 14 | 263 | 85 |
| 58 | 190 | 8.1 | 4640 | 78 | 1229 | 15 | 219 | 121 |
| 59 | 245 | 7.2 | 5440 | 179 | 1415 | 25 | 434 | 118 |
| 60 | 238 | 7.1 | 5250 | 175 | 1341 | 25 | 431 | 109 |
| 61 | 215 | 7 | 4920 | 154 | 1254 | 24 | 351 | 101 |
| 62 | 237 | 7.4 | 4990 | 159 | 1239 | 2 | 450 | 103 |
| 63 | 225 | 7 | 4910 | 154 | 1269 | 26 | 381 | 101 |
| 64 | 231 | 7.6 | 4360 | 118 | 1099 | 20 | 290 | 89 |
| 65 | 220 | 7.6 | 4990 | 153 | 1318 | 23 | 405 | 102 |
| 66 | 243 | 7.2 | 5330 | 182 | 1417 | 29 | 455 | 113 |
| 67 | 97 | 7.4 | 1772 | 84 | 51 | 0 | 43 | 25 |
| 68 | 92 | 9.1 | 1300 | 5.5 | 265 | 2.7 | 38 | 71 |
| 69 | 83 | 8.7 | 1000 | 0.8 | 2.6 | 0.5 | 30 | 59 |
| 70 | 53 | 7.6 | 350 | 0.8 | 10 | 0.5 | 12 | 10 |
| 71 | 94 | 7.3 | 816 | 18 | 194 | 1.9 | 40 | 8 |
| 72 | 96 | 7.6 | 1450 | 4.3 | 23.5 | 0 | 42 | 14 |
| 73 | 103 | 8.1 | 700 | 4 | 110 | 0.7 | 51 | 19 |
| 74 | 164 | 6.6 | 4120 | 75 | 564 | 10 | 184 | 98 |
| 75 | 156 | 7.7 | 3800 | 99 | 984 | 20 | 160 | 97 |
| 76 | 83 | 7.1 | 1800 | 8 | 98 | 7.6 | 30 | 31 |
| 77 | 183 | 7.6 | 4500 | 95 | 995 | 12 | 251 | 90 |
| 78 | 225 | 7.2 | 5090 | 140 | 1225 | 38 | 426 | 180 |
| 79 | 208 | 8.9 | 4890 | 129 | 1257 | 20 | 292 | 100 |
| 80 | 201 | 8.3 | 4750 | 126 | 1225 | 17 | 258 | 92 |
| 81 | 207 | 8.4 | 4590 | 117 | 1258 | 21 | 249 | 94 |
| 82 | 230 | 7.5 | 4680 | 131 | 1232 | 22 | 650 | 95 |
| 83 | 190 | 7.5 | 4500 | 74 | 1126 | 12 | 191 | 90 |
