# Supplementary material for: Reservoir temperature prediction based on characterization of water chemistry data—case study of western Anatolia, Turkey
Source: Sci Rep. 2024 May 6;14:10339. doi: 10.1038/s41598-024-59409-5 (PMC11074159; doi:10.1038/s41598-024-59409-5)
Supplement: Supplementary file 2 — Supplementary Information 2. [file 41598_2024_59409_MOESM2_ESM.docx]

# Appendix B. Equations of Na/K and SiO_2_ thermometers

| **Geothermometers** | **Equation（units:**$\boldsymbol{^{\circ}C}$**）** | **Reference** |
| --- | --- | --- |
| Na/K^1^ | $T=\frac{856}{0.857+lg(Na/K)}-273.15$ | Truesdell(1976) |
| Na/K^2^ | $T=\frac{1217}{1.438+lg(Na/K)}-273.15$ | Fournier(1979) |
| Na/K^3^ | $T=\frac{833}{0.780+lg(Na/K)}-273.15$ | Tonani(1980) |
| Na/K^4^ | $T=\frac{933}{0.993+lg(Na/K)}-273.15$ | S. Arnórsson (1983) |
| Na/K^5^ | $T=\frac{1319}{1.699+lg(Na/K)}-273.15$ | S. Arnórsson (1983) |
| Na/K^6^ | $T=\frac{1178}{1.470+lg(Na/K)}-273.15$ | Nieva(1987) |
| Na/K^7^ | $T=\frac{1390}{1.750+lg(Na/K)}-273.15$ | Giggenbach(1988) |
| SiO_2_^1^ | $T=\frac{1309}{5.19-lg(\mathrm{SiO}_{2})}-273.15$ | Fournier (1977) |
| SiO_2_^2^ | $T=\frac{1522}{5.75-lg(\mathrm{SiO}_{2})}-273.15$ | Fournier (1977) |
| SiO_2_^3^ | $T=\frac{1032}{4.69-lg(\mathrm{SiO}_{2})}-273.15$ | Fournier (1977) |
| SiO_2_^4^ | $T=\frac{1263}{5.32-lg(\mathrm{SiO}_{2})}-273.15$ |  |
| SiO_2_^5^ | $T=\frac{731}{4.52-lg(\mathrm{SiO}_{2})}-273.15$ |  |
| SiO_2_^6^ | $T=\frac{1000}{4.78-lg(\mathrm{SiO}_{2})}-273.15$ |  |
| SiO_2_^7^ | $T=\frac{781}{4.51-lg(\mathrm{SiO}_{2})}-273.15$ |  |
